# The International Medical Congress Muddle

**Published:** 1885-09

**Authors:** 


### THE INTERNATIONAL MEDICAL CONGRESS MUDDLE.

The following from the *British Medical Journal* of July 25th points plainly to what may be expected, unless some compromise is reached at the meeting of the committee to be held in New York on September 3d:

"The most recent advices from the United States have brought the startling intelligence that there exists in the American medical profession a very serious discord concerning the next International Medical Congress. We do not propose to discuss the etiology of this rupture, for it is quite enough to be called upon to face the fact that it exists. The fact is very grave. Its existence jeopardizes, if it have not already destroyed, the probable success of the forthcoming Congress. Certainly our brethren in the States cannot expect those who have already promised to attend and those who may expect to visit America at that time to work with enthusiasm in the preparation of any scientific contribution while those whom they propose to visit are divided, and while wholesale secession of the official executive and of well known persons nominated to high offices are announced. Nor do we consider it to be either our duty or privilege to suggest a remedy for this exceedingly unpleasant dilemma. It seems to be conclusive that the profession in America at this moment is hopelessly divided on the subject. Already a large proportion of the influential and active scientific men of Philadelphia, such as Bartholow, Weir Mitchell, Da Costa, H. C. Wood, Pepper, Leidy, Stillé, Parvin, and Goddell, and David Yandell, of Louisville, have publicly withdrawn from the organization of the Congress. A like number of distinguished men in New York, such as Loomis, Roosa, Jacobi, Mundé, Agnew and Emmet, have also either resigned or been dropped, and therefore will not co-operate with the present organization. The outlook, as the matter now stands, is not at all encouraging. One committee has reorganized the work of another up to the point near that of destruction. Moreover, the work of the present committee must be submitted to the American Medical Association in May, 1886; and no one can say to what extent it may also be either overturned or modified in such a way as to seriously impede the labor necessary to be performed before the meeting of the Congress in 1887. Altogether, the position is lamentable, and there is much fear that the acceptance of the invitation to meet in the States may be withdrawn, and the next meeting of the International Medical Congress be held in Berlin or some other great medical centre, pending the settlement of the serious dissensions among our brethren of the United States."

